# Adolescent sexual and reproductive health needs and utilisation of health services in the Bono East Region, Ghana

**DOI:** 10.1186/s12978-024-01822-0

**Published:** 2024-06-17

**Authors:** Joshua Okyere, Naomi Kyeremaa Yeboa, Charity Nikoi, Margaret Owusu-Amoako, Listowel Ferka, Anastasia Nurzhynska, Joshua Amo-Adjei

**Affiliations:** 1 Department of Population and Health, University of Cape Coast, Cape Coast, Ghana; 2 School of Nursing and Midwifery, Kwame Nkrumah University of Science and Technology, Kumasi, Ghana; 3 Department of Maternal and Child Health, School of Nursing and Midwifery, University of Cape Coast, Cape Coast, Ghana; 4 UNICEF Ghana Office, Social and Behaviour Change Communication Unit, Accra, Ghana; 5 UNICEF Kenya Office, Social and Behaviour Change Communication Unit, Nairobi, Kenya

**Keywords:** Adolescent, Sexual and reproductive health, Health service, Utilization

## Abstract

**Background:**

Adolescents in Ghana are vulnerable to unequal power relations at the personal, community and structural levels which in turn limits their opportunities in access to critical sexual and reproductive health information and services. There is therefore high unmet need for sexual and reproductive health (SRH) information and services and the Bono East region typifies this situation, recording some of the poorest SRHR outcomes among adolescents. We, therefore, aimed to investigate the SRH needs (unmet), behaviors and utilization of SRH services among adolescents in the Bono East region.

**Methods:**

Using a maximum variation sampling approach, this qualitative study conducted in-depth interviews and focused group discussions with adolescent boys and girls, parents, community leaders, and healthcare providers.

**Results:**

Our findings are presented under two broad categories: major SRHR concerns of adolescents, and perspectives about that influences adolescents’ utilization of SRHR services. Under the major SRHR need of adolescents, the following themes emerged: information and services on pregnancy prevention, menstrual hygiene management, availability of comprehensive abortion care services, and attitudes towards adolescent pregnancy. The perspectives about the factors that influence adolescent children were discussed at multiple levels: individual/personal. interpersonal and community/societal. At the individual level, limited understanding of adolescence/puberty, desire of adolescents to belong and misperceptions about contraceptives. At the interpersonal level, issues relating to technical capacity needs of service providers, disrespect exhibited by service providers, and parental failure were identified as influential factors. Then at the community/societal level, we identified structural constraints and compromised social safety concerns in accessing contraceptives and services.

**Conclusion:**

In conclusion, the findings from this study offer valuable insights into the complex landscape of adolescent sexual and reproductive health in the Bono East region. The implications for policy and practice are manifold, ranging from comprehensive education to addressing menstrual hygiene, involving parents, training healthcare providers, and promoting respectful care.

## Background

Adolescence is a transitional phase from childhood to adulthood [1]. This transitional period is often characterized by several storms that adolescents (i.e., 10–19 years) have to surmount. Such challenges include peer pressure, sexual maturation, desire, and curiosity to experiment with sexual activities [2]. In most cases, adolescents – particularly those in resource constrained settings – lack guidance and access to accurate, timely, and quality information necessary to shape their sexual and reproductive health (SRH) positively. The effects of these challenges reflect in adverse outcomes including, unintended pregnancies, unsafe abortions, sexual exploitations, sexually transmitted infections, repeat pregnancies, and intimate partner violence [3–6].

A report from the Guttmacher Institute indicates that in 2019 alone, there were nearly 21 million pregnancies among adolescents 15–19 years in low-and-middle-income countries (LMICs); half were unintended and 55% ended in abortions – mostly through unsafe methods [7]. The World Health Organization (WHO) also reports that in sub-Saharan Africa (SSA), the rate of childbirth among younger adolescents (i.e., 10–14 years) is approximately, 4.6 per 1000 women which is higher than the global 1.5 per 1000 women among the same cohort [8]. Adolescent SRH issues remain major public health concerns in many countries, including Ghana.

Adolescents in Ghana have high unmet need for SRH information and services. Evidence suggests that 82% of adolescent girls and 75% of adolescent boys lack comprehensive knowledge on HIV, with only 27% of sexually active adolescent girls utilizing modern contraceptives [9, 10]. In addition, child sexual violence Ghana; a study that revealed that approximately one-third of girls and around one in ten boys were coerced into first sex [11].and these are suspected to be underreporting of the exact magnitude of sexual and gender based violence against adolescents [12].

Both male and female adolescents continue to engage in risky sexual behaviors. Yeboah et al. [13] reported that 28.1% of adolescent boys engage in multiple sexual partnerships. Nearly 64.4% of adolescents engage in early sexual initiation [14]. Also, 21.4% of adolescents have been found to engage in sexual intercourse [15]. Adam et al. further report that 60.7% of adolescents who have sex when drunk do not use a condom [15]. Hence, increasing their risk of sexually transmitted infections and unplanned pregnancies.

Indeed, there is a preponderance of empirical studies in Ghana that have investigated SRH behaviours of adolescents and their utilization of SRH services [13, 14, 16]. However, these studies have mainly been quantitative [13, 14]. We therefore need deeper understanding of the nuances that characterize the SRH needs and service utilization of adolescents in Ghana. Furthermore, the Bono East, previously part of the broader Brong Ahafo region, exhibits concerning and enduringly poor adolescent SRH outcomes. For many years, the region has been one of the top three with the country’s highest prevalence of adolescent pregnancy [9, 10]. We, therefore, aimed to explore the persistent SRH needs of adolescents, availability and provisioning and utilisation of health services among adolescents in the Bono East region. Specifically, we addressed the following objectives:  

The paper is situated in the remit of the Social Ecological Model (SEM). We employed this framework as the conceptual framework because it allows us to understand how adolescent sexual agency is both influenced and constrained by the various levels within their ecological system [17]. These levels range from individual factors (e.g., knowledge and beliefs), interpersonal dynamics (e.g., peer and family influences) and community factors (e.g., gender, social and cultural norms), institutional arrangements (e.g., service guidelines; availability of services) to the national political and policy processes (e.g., policies) [17]. Essentially, the SEM suggests an overlap and interaction of various factors at different levels to shape adolescent SRH behaviors and outcomes.

### Policy context

To advance SRH services and outcomes among adolescents, Ghana adopted its first adolescent reproductive health policy (ARHP) in the year 2000. The ARHP envisioned increased age at first sex, reduction in child marriage by 37%, reduction in teenage pregnancy by 50%, and a reduction in abortion among young people by 50% by 2010 [18]. Further to this, the Ghana Health Service developed the adolescent health service policy and strategy (AHSPS) (2016–2020) [19]. The goals of the AHSPS included reducing adolescent mortality rates, ensuring that 90% of adolescents and young people receive vital information about health and services, facilitating a 90% knowledge level among adolescents and young people regarding SRH services and rights, substantially increasing comprehensive HIV knowledge among adolescent girls and young people from 20% to 60%, and aiming for at least 60% of adolescents accessing specialized adolescent health services by 2020 [19]. Additionally, the targets involved enhancing National Health Insurance card coverage among female adolescents aged 15–19 years from 52.7% in 2014 to at least 70% by 2020, and significantly increasing the utilization of modern contraceptive methods among females aged 15–19 years and 20–24 years, while decreasing the unmet need for family planning in these age groups [19]. Lastly, the AHSPS sought to increase condom usage among young males aged 15–24 with multiple partners from 34% in 2014 to 40% by 2020 [19].

## Methods

### Design and study setting

We employed the qualitative research approach to generate descriptive cross-sectional data. Bono East region, formerly part of the Brong-Ahafo region, was selected as the study area. Over the years encompassing the initiation of the Demographic and Health Surveys in the country, the region has consistently held the highest or second-highest rates of teenage fertility [9]. For instance, between 2008 and 2014, approximately 18% of adolescents aged 15–19 within the region had a live birth. Between this period, the region's ranking in terms of teenage fertility was second only to the Central and Volta regions [9, 15]. Based on this premise we conducted the present study in districts within the Bono East region.

### Study population

The study targeted multiple stakeholders. These included adolescent boys and girls, community and religious leaders, parents, healthcare workers attending to adolescents SRH needs, and education workers (i.e., teachers and school health educators).

### Sampling

For the adolescents, a maximum variation sampling approach was used. This involved the deliberate sampling of adolescents with different characteristics (e.g., age, gender, urban–rural residence, and level of education). As a type of purposive sampling technique, maximum variation sampling ensures that the relevant people participate in a study and ensures the sample’s diversity [20]. Parents and community leaders were purposely selected with the assistance of community mobilizers, such as local district assembly delegates. The study team provided an explanation of the nature, objectives, and characteristics of eligible participants to mobilizers/facilitators, who subsequently facilitated the recruitment of participants by the fieldworkers.

### Inclusion and exclusion criteria

To be eligible to participate in the study, adolescents were expected to be between 15–19 years and be usual residents of the study area. Moreover, the adolescent must have the mental capacity to assent or consent. Healthcare workers were considered eligible to participate in the study only if they provided direct SRH services to adolescents (i.e., contraceptives, family planning, safe abortion services, SRH information, and education). Consequently, younger adolescents (i.e., 10–14 years), those who could not assent, and those whose parents did not consent were excluded from the study. In addition, community leaders, religious leaders, and parents who did not identify as usual study area residents were precluded.

### Data collection

Prior to the data collection, we recruited three qualitative research assistants who were responsible for conducting the in-depth interviews (IDIs) and focused group discussions (FGDs). These research assistants had a minimum of bachelor’s degree in population health, and at least two years’ experience conducting qualitative research. Notwithstanding, the last author (JAA) who was the supervisor in this project organized a five-day training to reorient the research assistants, and get them informed about the study objectives, methodology, and instrument (see Supplementary file 1). Additionally, the training involved role plays to simulate how to correctly ask the questions in the local language (Twi). We conducted in-depth interviews (IDIs) and focused group discussions (FGDs) with the study participants. A total of 26 IDIs were conducted using a semi-structured interview guide. Among them were adolescents (*N* = 16), comprising an even distribution of eight girls and eight boys. Additionally, parents (*N* = 5), including three mothers and two fathers, were engaged in the interviews. Community leaders (*N* = 5), consisting of two females and three males, participated in the IDIs. There were three FGDs with the adolescents. This involved a total of 23 participants who were split by gender, with seven boys and 16 girls (8 in each group) contributing to the discussions. It must be noted that the participants of the FGDs were different from those who were interviewed in the IDIs. Except for the IDIs with adolescents conducted exclusively at their homes, the remaining interviews were conducted at the home, offices, and other community spaces where participants’ and interviwers’ privacy, confidentiality, and safety could be maximized. Each IDI and FGD was audio recorded after having obtained both verbal and written permission from the participant. The interviews were conducted between November and December, 2022. On average, the IDIs lasted about 45 min whereas the FGDs lasted 80 min. All FGDs as well as 23 of the IDIs were conducted in Twi, while three IDIs were conducted in English. This was solely based on the preference of the participants.

### Data analyses

The recorded interviews and FGDs were transcribed and imported into QSR NVivo-12 for data management and analysis. The analysis was done following Clarke et al.’s [21] six-phase analytic strategy. In the first phase, all audio recordings and transcripts were repeatedly reviewed to immerse ourselves in the data. This process allowed us to familiarized ourselves with the participants' voices, viewpoints, and experiences. JO and JAA performed a line-by-line coding of the transcript to identify significant data segments related to adolescent SRH needs and service utilization. This process was first done inductively to allow the codes to organically emerge from the data. After this, a deductive coding was done, taking into consideration the socio-ecological model. The assigned codes were collated into potential themes in the third phase by grouping related codes. Through constant comparison and discussion among the research team, preliminary themes were identified, allowing for the exploration of patterns and variations in the data. Where there were contrasting perspectives about a theme, the entire research team discussed it until an agreement was reached. The identified themes were then critically reviewed and refined. The process involved assessing the coherence and distinctiveness of each theme and ensuring that they accurately represented the participants' perspectives. After a thorough review, the refined themes were defined and given descriptive names that encapsulated their content. Each theme was accompanied by a clear explanation of its relevance to the research objectives. The final step involved synthesizing the themes and their respective sub-themes into a coherent narrative [21]. This narrative provided a comprehensive account of the stakeholders' perspectives. Exemplar quotes were selected to illustrate the themes, enhancing the credibility and richness of the findings.

### Rigor and trustworthiness

The triangulation of data sources including IDIs and FGDs with adolescents, parents, and community leaders enhanced the comprehensiveness of the findings and reduced the potential for bias arising from a single data type [22]. Regular peer debriefing sessions were held among the research team members. These sessions provided a platform for open discussion, critical feedback, and exploration of differing viewpoints, thereby enhancing the researchers' awareness of their own biases and aiding in the refinement of the analytical process. To enhance the confirmability of the study, an audit trail of the interview guide, informed consent form, and assent forms were documented. The inclusion of direct quotes and vivid descriptions strengthened the credibility of our findings [23].

### Ethical considerations

Ethical approval was obtained from the Ghana Health Service Ethical Review Committee [ID: GHS-ERC-013–09-22]. All the methods and procedures are in line with the Declaration of Helsinki [24]. Before conducting fieldwork, the research assistants were trained on the ethical principles of respect, beneficence, and justice. The training emphasized the corresponding ideas of autonomy and self-determination, namely the right to access information, voluntary involvement, avoidance of exploitation, privacy, and secrecy. Additionally, we obtained both oral and written consent to participate in the study. In the case of individuals under the age of 18, we sought consent from the parents or legal guardians, followed by the child's assent. In the case of emancipated minors, i.e., teens who had assumed parental roles, we obtained personal consent. All the research assistants signed a non-disclosure agreement. The audios and transcripts were anonymized and encrypted with a password to prevent unauthorized access.

## Results

**Fig. 1:**
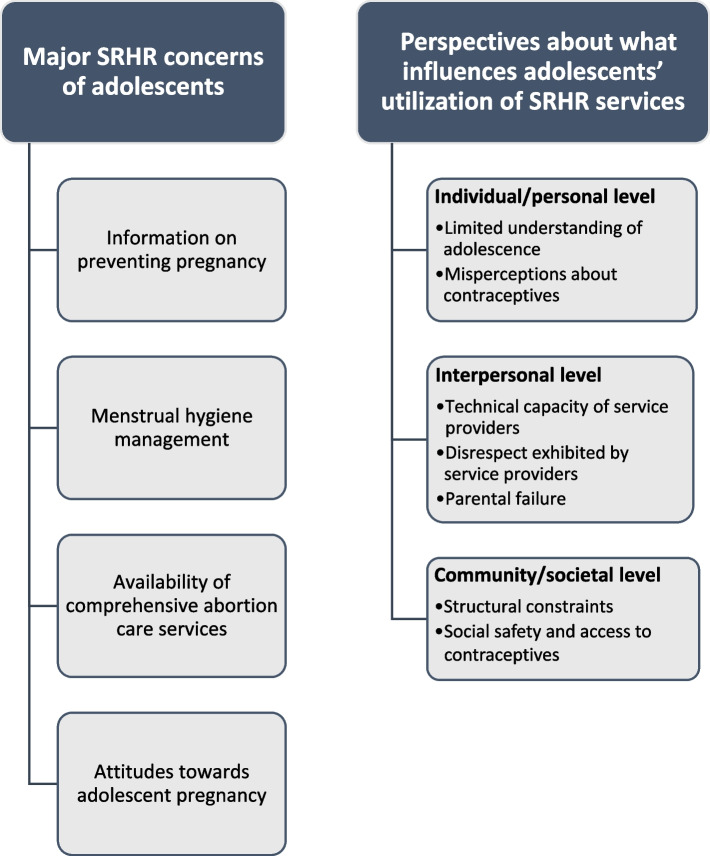
Thematic map of themes embedded within the SEM

Our findings are presented under two broad categories: major SRHR concerns of adolescents, and perspectives about what influences adolescents’ utilization of SRHR services. Under the major SRHR concerns of adolescents, the following themes emerged: information on pregnancy prevention, menstrual hygiene management, availability of comprehensive abortion care services, and attitudes towards adolescent pregnancy (Fig. 1).

The perspectives about the factors that influences adolescents’ utilization of SRHR services was presented in three themes as informed by the SEM. At the individual or personal level, limited understanding of adolescence, yearning for a sense of belonging and misperceptions about contraceptives emerged as the key factors that influences an adolescent’s utilization of SRHR services. At the interpersonal level, issues relating to technical capacity needs of service providers, disrespect exhibited by service providers, and parental failure were identified as influential factors. Then at the community and societal level, we identified structural constraints and compromised social safety to access contraceptives as the key influencing factors.

### Major SRHR concerns of adolescents

#### Information on preventing teenage pregnancy

In response to a question on adolescents most pressing SRHR concerns in the communities, participants unanimously – (parents, community leaders, adolescents, and health workers) mentioned pervasive unmet need for information and education on how to prevent early and unintended pregnancy. According to participants, providing adolescents with preventive services and products could help minimize the widespread occurrence of teenage pregnancies in the region. Some excerpts are provided as illustration:

When you look at it, what they need is adolescent groups where they would get to learn about the dangerous consequences of teenage pregnancy (Adolescent SRH Provider, 43 years, Techiman North)

I know some of the health needs of adolescents. They need condoms to protect themselves from diseases and pregnancies. Because diseases and unintended adolescent childbearing are too prevalent in the Techiman North. So, if they can supply youths/adolescents with condoms, it will be better (Adolescent Boy, 16 years, Techiman North)

#### Menstrual hygiene management and products

The next frequently mentioned SRHR need of adolescents was menstrual hygiene education and products. This was pervasive in the narratives of adolescents – both boys and girls, and health providers. Participants intimated that many girls lacked comprehensive knowledge of menstrual hygiene and sanitation. One health provider proffered:

They don’t have much knowledge about their personal hygiene especially those in their menstruation stage, how to take care of themselves during that stage is a problem. (Midwife, Kintampo South)

An adolescent boy added:

Some of the girls do not know their menstrual cycle; sometimes even in public places, including classroom, the menses come when they are unprepared. So, they need education. (Adolescent Boy, 17 years, Offuman, Techiman North)

Also, the accounts revealed that several girls are not able to afford menstrual hygiene products, and this was mentioned as an underlying cause of transactional sex which contributed to unintended pregnancy and STIs among adolescents.

The girls need menstrual pads. During menstruation, we need pads. Some girls do not have money to buy a pad during their menstruation and that makes them go out to look for money to buy these things. (Adolescent Girl, 15 years, FGD participant, Kintampo South).

#### Information on, and access to safe abortion care

In the narratives are accounts pointing to a need for information and education on safe abortion and post-abortion care services. These views emanated largely from adolescents and health providers. From providers’ accounts, safe abortion services were available in some of the facilities, while others did not have the capacity to provide them. The providers generally supported the need for making safe abortion services available to adolescents, arguing that without such services, adolescents may resort to unsafe methods to terminate unintended pregnancies. The providers' narratives indicated that some had either treated or referred adolescents with post-abortion complications. A health provided discussed:

Yes, what happens is that we have trained professionals so when girls come here, we refer the girls to them. At the maternity they have trained doctors who provide comprehensive abortion care so when they come here, we refer them. (Health provider, Atebubu)

Some adolescent participants knew or had heard of some stories of post-abortion complications in their peer circles. This was attributed to limited-to-no information and knowledge of where to access safe abortion services. Adolescent participants mentioned different home-made methods for inducing abortion. Participants who discussed these home-made therapies appeared confident about the efficacy of the same to terminate an unwanted pregnancy. A participant illustrated.

We have a way of getting rid of pregnancy; you just buy Guinness so I will tell her to drink Guinness. Guinness and akpeteshie are quick solutions to abortion (Adolescent Boy, 19 years, Jema, Kintampo South)

The use of such unsafe procedures/methods was attributed to the cost of safe abortion services, often beyond the means of adolescents. Health providers and adolescents affirmed:

Even with comprehensive abortion, providers are asked to charge a token fee – between Ghana Cedi 300 – 600^14^ but in these communities that is a huge cost that most cannot afford so they will try to do some concoction or take something to end it [pregnancy] at home instead of coming here. (Male Health Provider, Atebubu)

You can go to the hospital to abort it, but there is no way that aborting a baby in the hospital is free. The herbs are cheaper. You can spend over GHs 500 ($50), so it can be due to money, the amount of money she has at that time might be small. And the person doesn’t want the child but cannot afford to go to the hospital. (Adolescent Girl, 15 years, Breman, Kintampo South).

#### Attitudes towards adolescent pregnancy

Attitudes manifest in three forms: cognitive, emotional, and behavioural disposition towards a phenomenon. Attitudes towards adolescent pregnancy are analysed within these constructs. In terms of cognition, the data shows that adolescent pregnancy is generally viewed as a negative experience in the life of an adolescent. This view is shared unanimously among adolescents, parents, community leaders, and healthcare providers. The disruptive effects of adolescent pregnancy on schooling potentials were frequently mentioned by participants. Some adolescent girls elaborated in a FGD:

If the boy gets you pregnant, as for him, the parents will allow him to go to school. But for the girl, they will not allow her. The family will be insulted that they have allowed their daughter to get pregnant. (FGD participant, 16 years, Asueyi, Techiman North).

All the stress will be on the girl and the girl cannot go to school with the pregnancy … after delivery, she may not go back to school again and her education will end. (FGD participant, 17 years, Asueyi, Techiman North).

Adolescents, especially girls, who were able to avoid unintended pregnancies were commended and admired greatly. In FGDs with adolescent girls, they discussed how community elders and parents respected adolescent girls who did not become mothers during the adolescence period. Girls in FGDs shared the following popular attitudes towards adolescents who are not mothers.

They are happy with them (non-adolescent mothers) when they see them. In addition, they say things like you have kept yourself well. And sometimes your community head can complement you and say things like; you have done really well; you did not expose yourself to men. (FGD participant, 17 years, Kintampo South).

R3: When that happens the elders, respect you. They say things like, this girl is a good girl, she was not promiscuous, so she has been able to progress in education. (FGD participant, 17 years, Kintampo South).

## Perspectives on drivers of adolescent pregnancy, SRH services availability and utilization

### Individual/personal level

#### Limited understanding of sexual and reproductive physiology

At the individual level, some of the participants argued that many adolescents lacked comprehensive understanding of the adolescence period, especially the physical, biological, and emotional changes that occur during this period. Due to this lack or limited knowledge of the body’s mechanisms and evolving sexual development, some engaged in unprotected sex without mindful of the risks unintended pregnancy and STIs.

As a growing boy or girl, sometimes you get some ‘feelings.’ The desire for sex is innate; so, emotions are also a cause. (Adolescent Boy, 16 years, Offuman, Techiman North)

Parents and community leaders on the other hand argued that low risk perceptions associated with some sexual activities (e.g., kissing and fondling), which could trigger penetrative unprotected sexual intercourse, was a major challenge for adolescents. However, they recognised that emotional and biological changes associated with adolescence could increase the desire for sexual intercourse and in the absence of protection, lead to pregnancy. This group of participants did not attribute adolescent pregnancy to any socioeconomic factor(s) other than sexual maturation. A community leader concurred with this assertion:

I think naturally there are these feelings they get in their bodies that prompts them to do so (have sex). (Community Leader [Pastor], Kintampo South).

#### Misperceptions about contraceptives

The data also revealed negative perceptions around contraceptives, which discourages some adolescents and young people from use. For instance, there was a widespread belief that using contraceptives in adolescent years could cause infertility in the the adult years. From the data, some adolescents and parents expressed strong sentiments against use of contraceptives for fear of infertility. An example of such view is:

I would rather get pregnant than use contraceptives because those medicines can cause side effects for you. When you use it for so long there will be a time you will want a baby and you will not get. (Adolescent Girl, 18 years, Atebubu, Atebubu-Amantin District).

### Interpersonal level

#### Technical capacity of SRHR providers

Utilization of health services is a function of capacity of providers to offer what clients require. In situations where users have little confidence in the ability of providers, utilization is likely to be affected. We asked providers the types SRHR services they did not have capacity to provide. Principally, comprehensive abortion care and minimally invasive family planning methods were frequently mentioned as can be seen from the extracts below:

I cannot provide some family planning methods. I can give the injection but the other procedures, implants, and IUD, I cannot do it. (Midwife, Offuman, Techiman North)

I am not that good in family planning and so it is not everything that I can do, I must call someone to assist me so that if I am okay, when someone comes hereandIamtheonly one here I can do it … with the abortion care, I have no skills at all. (Midwife, Kintampo North).

#### Parental capability to provide resources and information/education

On the parental side, participants shared that adolescents who became pregnant often did not have the requisite parental care. Adolescents who participated in the study discussed that parents did not sufficiently connect with their children, especially girls. Parental failure appeared in different ways: 1) inability/failure to provide necessities (e.g., menstrual hygiene products and school supplies) for their children, and 2) failure of parents to provide children suitable guidance on sexual behaviours (i.e., home-based sexuality and reproductive health education). For many adolescents, there is no motivation to discuss their SRHR issues with their parents for fear of being judged as spoilt/bad. One boy intimated:

Because if you discuss such things (sex-related) with your parents, they will say you are a bad boy. So, we discuss it with our friends. Not our parents. (Adolescent Boy, 18 years, Offuman, Techiman North)

Another form of parental deficiency that adolescents lamented about was maltreatment and low parent–child connectedness or quality of relationship between parents and adolescents. For poor parent–child relationships, it was viewed as a push factor for adolescents to seek affection, love, and belonging through sexual relationships. When faced with an unconducive home environment, some adolescents may find solace in sexual relationships, which could result in unintended pregnancy. A participant noted:

Some of the guardians make the children uncomfortable. They are always in fear of these guardians.… You can clearly see that these children are unhappy all the time. Even when they do not intend to be in a relationship with the opposite sex, they end up being in a relationship. If they do not know how to protect themselves, they become pregnant in the end. (Adolescent Girl, 15 years, Kintampo South)

Discussions with some adolescent mothers showed that while none deliberately wanted to become a mother, the sexual intercourse that led to the pregnancy was intentional and that they were often led by some push factors. In many instances, they had some material and financial needs that were not provided by their parents. Adolescent mothers with such experiences blamed their parents’ inability or failure to adequately provide for their needs which pushed them into transactional sex and pregnancy consequently. A 17-year-old mother in Atebubu described:

When I got pregnant, I didn’t blame myself because when I am going to school, and I ask my mother for money she doesn’t mind me.

#### Disrespect and abuse of adolescents: when is it justified?

Further to these, we investigated the circumstances under which providers may be justified to disrespect and abuse adolescent clients. Two key circumstances emerged: when clients do not comply with appointments and instructions and when they refuse effective treatment.

On non-compliance to appointments and treatment instructions, – adolescent girls, mainly, justified abuse and disrespect. They viewed health workers as authorities in issues of health care and viewed non-compliance to well-meaning instructions as grounds for adolescent clients being treated harshly. Extracts from two adolescent girls are illustrated here:

Maybe you are asked to come for a scan at 3 months and you don’t go, as you don’t go for the scan it will be difficult for them to know what is going on, she will not be happy with you because you didn’t follow her orders. Let’s say you have all been asked to come to the facility at 7:30 and at 10:00 you are now coming; it angers the health workers. (Adolescent Girl, 18 years, Jema, Kintampo South).

It is not about their (health workers) attitude; when you are pregnant, you need to buy certain things for your delivery so if you come and you don’t bring anything at all then what will the put the baby on? You will give them the opportunity to mistreat you if you don’t do what is required of you. That’s what I am saying. If you don’t take the things needed for delivery and you also do not take money, they ask you to do this, you don’t do it, they give instructions, and you don’t follow so that is what will happen. (Adolescent Girl, 18 years, Atebubu).

Adolescent participants also argued that an adolescent who persistently refused a known effective treatment could be threatened or disrespected/abused. This view was underpinned by participants' notion that health providers were only interested in the wellbeing of their clients. Consequently, providers would be justified to threaten or abuse clients who do not adhere to specific therapeutic instructions. One provider narrated an experience:

Yes, I remember that I delivered one girl and after delivery I had to expel the products and she kept shifting herself. At a point she told me to leave it like that because it was paining her and I told her that if I leave it and she goes, it will still bring you back so for something like that you still have to do it. (Midwife, Kintampo South).

Some adolescent participants also discussed that in cases where providers judged a particular treatment to be the best option and there was threat to lifer, the provider could compel the client, failing which they could be called to undertake more difficult services. The following excerpt provide some context:

Yes, madam, for instance, family planning. A nurse might recommend it because they perceive that the girl cannot abstain from sexual intercourse, yet she is refusing to accept family planning. You (the health provider) must force her to do the family planning else she will get pregnant and come with another pregnancy. (Adolescent Girl, 15 years, Jema, Kintampo South).

### Community and societal level

#### Social safety, moral hazard and access to contraceptive education and services

The data generally showed acceptance to providing SRH education to adolescents. For instance, many participants including community approved educating adolescents on contraceptives. However, some participants discussed the need for age-appropriateness in SRHR education and services. For instance, on contraceptives and specifically condom education, most parents and community leaders (CLs) disapproved condom education for early adolescents (10–14 years). It was described as a moral hazard or perverse incentive to early adolescents. They argued that early adolescents lacked the maturity to process the information and education within the right context. They feared that early adolescents may experiment with what they have learned about contraceptives and condoms. However, CLs and parents supported educating older adolescents (15 – 19 years) on contraceptives, as they were deemed old enough to appreciate that form of education from different perspectives and contexts. An interaction with a male Pastor in Kintampo South is reproduced here:

Moderator: Now, we want to look at some of the SRHR services that will be accepted by leaders in this community. Will the community find it acceptable for children aged 10 to 14 years to be given sex education?

Participant: I will give it 100% support. I agree and we must accept it because we want to prevent this menace (adolescent childbearing). So, if this is going to help then I agree to it.

Moderator: What about education on contraceptive uses (e.g., condom) for children aged 10-14 years in the community?

Participant: No as for that one I don’t think it will help. When we teach them those things then we are telling them to go and do it. Rather, we should teach them to abstain. So, in my view we should not teach them those things.

Moderator: What about those who are 15-19 years?

Participant: I think 15-19-year-olds are of age to know those things. They could be taught that if they are not able to take care of a child, use these things (contraceptives) to protect themselves. But the smaller ones should be taught to abstain from these things [sexual intercourse].

However, while there was support for the education, providing the products (e.g., condom) was rejected, again, citing moral hazard. A parent with an adolescent child remarked:

When you come to teach them (adolescents) without giving them the condoms, that will be fine. However, when you expose them to condoms as children, they would want to try. (Mother, 43 years, Kintampo).

On the other hand, some CL rejected a universalist approach in providing SRHR services and education to adolescents, arguing that not all adolescents may be able to abstain from sexual activities. In effect, some CL had made personal efforts in making condoms available to adolescents after offering them sexuality education. One noted:

For me, after taking them through sexuality education I request for condoms and I tell them, if you can’t abstain and you don’t have money to buy certain things, I advise that you come so that we will get you some to protect yourself. (Community Leader, Kintampo North).

Among health workers and adolescents there were no exceptions to when adolescents could learn and access contraceptives. They asserted that abstinence and protected sex must not be treated as mutually exclusive; none must be prioritized over the other. This was needed to safeguard all adolescents due to the diversities of sexual responses during the maturation process. Some illustrative excerpts are:

I will say yes. We are all not the same, someone will listen, someone will not so by all means, she will have sex so if you want to have sex, the protective thing is to use condoms (Adolescent Girl, FGD participant, Kintampo South).

Some people will listen, and some will not. Now there are many diseases so if they use condoms, they can protect themselves from diseases. And she will not get pregnant (Adolescent Girl, FGD participant, Kintampo South).

With this recognition, health workers reported using a dual approach in working with adolescents—abstinence, and protection through contraceptives (including condom for dual protection against unintended pregnancy and STIs). One health provider remarked:

We teach them about abstinence. We also talk about protection against pregnancy and STIs. We try to make it clear to them that if they want to have sex, they must ensure that they are protected. (Health Worker, Kintampo South).

Adolescents, however, discussed the merits of knowing about methods of contraception and being able to access them. Adolescents generally viewed contraceptives as important in preventing adolescent pregnancy. They further argued that complete abstinence was not a realistic expectation during the adolescence period. Making contraceptives/family planning methods readily available for adolescents was described as a need. Participating adolescents also mentioned the added benefits of some contraceptive methods in protecting against STIs. Some illustrative quotes are below:

It is a concern. Maybe there is no money at home but as an adolescent the desire for sex is real, so you must be careful, you must protect yourself by using condoms. Family planning alone is not going to protect you from sickness. As a man you must protect yourself so that if the girl has any diseases, you will not be affected. (Adolescent Male, FGD participant, Atebubu).

On access to contraceptives, two views were noted from the data. One being that accessing contraceptives from pharmacies and chemical shops was less challenging. Adolescents who had accessed contraceptives from pharmacies and chemical shops expressed satisfaction with the services they received. Largely, these providers – mainly private—were described as non-judgemental in providing services. To some of them, the services they received were beyond their anticipation, as opposed to how adolescents were treated at some health facilities. That is participants shared that prior to their own personal experiences in accessing contraceptives, they worried about being judged and possibly turned away by providers. However, what they experienced was positive as shared below:

From the pharmacies I have gone to, it was normal to my surprise. The pharmacists didn’t raise an eyebrow; they don’t care about selling contraceptives to you (me). (Adolescent Boy, 19 years, Tuobodom, Techiman North).

However, accessing contraceptive/family planning products/services from health facilities was described as challenging on account of social safety. Here, the view was that some family planning outlets provided little-to-no privacy and confidentiality to patrons. Consequently, adolescents found it uncomfortable to access contraceptive/family planning services from health facilities. Adolescents and providers shared similar perspectives. The account below is an illustration:

I buy it (contraceptives) from the chemical shop. When I am entering a pharmacy or chemical shop, you don’t know what medicine I am going to buy; maybe it is my mother who has sent me to buy medicine for her. However, in the clinic, they have a particular room they do the family planning and once you enter that place everybody will be watching you and by the time you get home, your mother is even aware of what you went there for (Adolescent Girl, 18 years, Offuman, Techiman North).

Some adolescents reported being questioned by adult providers about the purpose of contraceptives they were purchasing. Even though providers are professionally required to provide guidance on dosage of medications/drugs, the manner and tone of inquiry can create discomfort for clients and discourage patronage of services. An adolescent recounted past experiences in line with this:

When I go there, and a male is not there how will I buy it? If there is an elderly person there, how can I buy it? They will ask what I am going to use it for at my age. I remember the last time someone asked me to buy a contraceptive for her, she wrote it on a piece of paper. When I got there, they were asking me what I was going to use it for. (Adolescent Girl, 18 years, Atebubu, Atebubu-Amantin District).

#### Structural constraints

Among health providers, the data revealed structural barriers that were similar across all facilities. Among them was lack of adequate infrastructure, (i.e., the quantity and location). In the view of providers, the available infrastructure compromised the privacy of adolescents who needed certain SRH services. Some of the providers recounted instances where adolescents would make inquiries about certain services outside of facilities but would decline coming to the facilities for fear of being noticed by family relations or other acquaintances. An illustrative excerpt is shared here:

Last time I met a girl, I referred her, but she told me if she comes, her aunt who works here will see her. Here, the clinic is very open. Everyone can see you. And if the person is not pregnant, why will she enter a maternity building? So, if someone is sitting at the out-patient department (OPD), they can see you and so by the time you get home, your mother will start asking you questions. Where did you go to, that is what they do so it makes the adolescent reluctant to come. (Health Provider, Techiman North, Tuobodom)

Also, the accounts showed that some basic surgical equipment for family planning, safe termination of pregnancy and management of post-abortion complications were inadequate. This often led to delays in providing adolescents timely services; the turnaround time to transition from one client to other was longer as they had to make allowance for disinfection before re-using equipment and materials. A provided recounted:

We have the instruments we use but it is not many so after using it for this person you must disinfect it before you come and use it for another and that makes the work someway (awkward). (Midwife, Kintampo)

Another structural issue that healthcare providers faced which affected service delivery to adolescents is the lack or limited number of teaching and learning materials for adolescents. The expectation was that at adolescent corners, providers would have the materials and the equipment to support learning and interactions among adolescents. However, these learning materials were limited in all the facilities studied. This in turn discouraged many adolescents from patronizing the adolescent corners. A provider elaborated as follows:

If we have maybe games like ludu, dummy, cards, adolescent games…when those things are available, it will really help. … those things that will make people happy to come here always, if they come here and things are not here, they will not be happy to come here but if all those things are available then they will be happy to come here. (Health Provider, Atebubu, Atebubu-Amantin).

#### Satisfaction with maternal health services for adolescents

Two contrasting perspectives were noted about adolescents' satisfaction with maternal and child health services. On the one hand, adolescent mothers gave positive testimonies about the quality of provider–client interactions. They described providers as friendly, warm, and showed interest in their welfare. Adolescent mothers recounted positive stories about being given nutrition counselling and the importance of attending clinics on scheduled dates. Two adolescents narrated their recent experiences as follows:

They weigh the baby and teach you how to care for the baby. They tell us that every month we should bring the baby for weighing for them to take care of them. The nurse that attended to me when I had my first child did not mistreat. They will take care of you in the sense that they will direct you to where you are supposed to go, if you need a scan to show how the baby is lying in your womb, they will ask you to go for a scan and they will interpret the results to you. They take good care of you here and if you don’t understand anything and you ask them politely, they will explain things to you. (Adolescent Mother, 19 years, Atebubu).

They sit all of us down and talk to us about what foods are more nutritious for us currently (during pregnancy and breastfeeding). (Adolescent Mother, 18 years, Jema, Kintampo South).

Notwithstanding the positive accounts of some adolescent mothers, others held contrary perspectives. This group of participants had heard many stories abuse and disrespect of peer adolescent mothers at health facilities for different reasons. Such stories made some sceptical about accessing SRH services completely. And what was particularly striking about some of these stories is that they were narrated by adolescent boys, as illustrated in the following:

Because they are young, health workers sometimes tell them that they have rushed into motherhood and how they are treated before they get the care, they want is not the best. They get the treatment alright, but the adolescent mothers are shy to go to the facility because they are shy, and they will also be mistreated. (Adolescent Boy, 19 years, Tuobodom, Techiman North).

An adolescent mother also shared a third party’s account:

I know someone who from the time she was discharged with her baby, she has never been to the hospital for weighing. If you also don’t buy the things, they have prescribed for labor they will shout on you. They will ask you to bring Dettol, rubber (mackintosh) pad and other things so if you don’t take them, which patient’s birthing accessories do you expect to use at the hospital? (Adolescent Mother, 18 years, Atebubu).

## Discussion

This qualitative study sought to unearth the sexual behaviors of adolescents, demand for, and provision of quality, relevant, and responsive SRH services to adolescents in Bono East region from the perspective of multiple stakeholders. Overall, four themes revolving around the perceived major SRH concern of adolescents, existing notions of the drivers of adolescent pregnancy, barriers to the utilization of adolescent SRH services, and an exposition on disrespectful SRH care emerged.

The finding on adolescents’ need for information about pregnancy as a major SRH concern is not surprising as it resonates with previous empirical evidence from Rwanda [25] and Ghana [26] that highlights the critical role of adolescent pregnancy prevention information. Our findings also align with a qualitative study conducted in Nigeria [27] which found the stakeholders’ perspectives about adolescents’ SRHR needs to include information on pregnancy prevention. As posited by the participants, access to adolescent pregnancy information goes beyond educating the adolescent about the dangers of pregnancy at this phase of their lives; it also includes providing comprehensive information about contraceptives including condom use and linkages to service providers. The findings affirm the persistent challenges of risk-informed sexual and reproductive health education by adults and parents which may not reflect adolescents realities and experiences. While adult experiences and perspectives can be profoundly important for adolescents safety and wellbeing, this must bear appropriate balance with adolescents biological, social and emotional dispositions to ensure a win–win outcomes.

In addition to adolescent pregnancy information, the study revealed that menstrual hygiene management and access to menstrual hygiene products was another significant concern for adolescent girls. Knowledge of the menstrual cycle; Similar patterns have been documented in Northern Ghana where 53.6% of adolescent girls reported poor knowledge about menstruation and how to prepare themselves for their period [28]. This lack is often exacerbated by the adolescent’s inability to afford the cost sanitary pads as documented previously [29]. The inability to afford sanitary pads not only hampers their ability to manage their menstrual hygiene effectively but also contributes to a range of negative consequences, including increased vulnerability to sexual exploitation which can lead to adolescent pregnancy. An emerging SRHR advocacy in Ghana is urging the government to remove taxes on menstrual pads to make it accessible to girls and women. In a recent study on child sexual exploitation in Ghana [30], it was documented that the inability of some girls to afford menstrual hygiene products contributed to sexual exploitation. Similar findings have been reported a qualitative study conducted in Kenya where adolescent girls highlight the difficulty in affording sanitary pads; this situation forces them to resort to transactional sex [31]. It is therefore important that the economic barriers are removed to enhance sexual and reproductive health and rights of adolescents, especially those from disadvantaged backgrounds. Although service providers highlight the availability of comprehensive abortion services in some facilities, the findings suggest that a section of the participating adolescents were unaware about these services. For some of those aware, they spoke favourably about local concoctions on account of high cost of facility-based services compared to home-made preparations. Kyilleh et al.’s [32] and Gbagbo [33] earlier studies have documented similar observations. This underscores a need to reassess Ghana’s current health financing regime to consider subsidizing comprehensive abortion care, especially for adolescents.

Parental capability to provide reliable, appropriate cognitive, affective, and material resources for adolescent children, especially girls emerged. This notion was driven by two key factors. First was the unwelcoming attitudes of parents to sexual communication and socialization of their adolescent child. The inability or unwillingness of parents to be involved in the sexual socialization of their adolescent children creates a lacuna, which can be exploited by both peers and older people for sexual gratification [25]. In such instances, the quality, accuracy and age-appropriateness of the SRH information received from these sources may not be reliable. Consequently, the wellbeing of many adolescents could jeopardised due to unsafe sexcal experimentation, multiple sexual partnerships and sexual violence/abuse [32]. Consistent with Morgan et al.’s study [34], we found that concerns about parental failure, in the sense of inability or unwillingness to provide for the adolescent’s socio-economic needs, was viewed as a major driver of adolescent pregnancy. Within the larger context of male-provider norm in Ghanaian communities, adolescent girls may seek or accept sexual offers in exchange for material goods, an extensively documented phenomenon in Ghana and SSA generally [35–39]. With parents and communities sometimes resisting school-based sexual and reproductive health education, the emerging documentation of parenting guidelines [40] becomes more urgent. Making these resources available (including in local languages) may be promising in the interim while efforts are underway to gain political concurrence for scale-up of formal in-school and out of school sexual and reproductive health education/interventions [41].

Similar to a study conducted in Uganda [42], the lack of technical capacity to provide certain SRHR services (e.g., abortion services) and medium to long-acting family planning commodities was evident in this study. Technical capacity to provide these services extend beyond medical knowledge; they encompass a holistic understanding of adolescent needs, cultural sensitivities, and ethical considerations. Training healthcare professionals to offer a range of SRH services, including abortion care and diverse family planning options, is essential to bridge this gap and address the specific requirements of adolescents. Our study also highlights the existence of structural constraints in relation to space that support the privacy of the adolescent, and the availability of sufficient SRH teaching and learning materials; these are consistent with extant literature [23, 26, 28]. Addressing these structural deficiencies will boost the confidence of the adolescents and build their trust to seek SRH services and information.

The final aspect of our study was to understand the nuances of respectful and dignified SRH service provision to adolescents. We found that adolescents perceived health service providers to be justified in exhibiting acts of disrespect and intimidation when they [adolescents] were non-compliant to appointment and treatment instructions. Usually, in weak health systems, the wide power disparities between providers and their clients tend to undermine the entitlements of patients to receive rights-based services. In such contexts, it requires highly ethical providers to avoid abusing their fiduciary obligations to their clients. This is particularly the case as clients may appear vulnerable, regardless of the prevailing human rights ethos. For adolescents seeking SRHR services, including maternal and child health, these power imbalances may be more acute given the high stigma around adolescent children. This can explain adolescents internalising disrespect and abuse as normal even they are not.

### Policy implications

The findings emphasize the critical need for comprehensive sexual health programs that cater for adolescents' diverse needs. It is imperative to provide adolescents with accurate information about pregnancy prevention, contraceptives, menstrual hygiene management, and safe abortion care, including where to access services. This information should be age-appropriate, accessible, and presented in ways that resonate with adolescents' experiences and concerns. Furthermore, the role of parents in adolescents' SRH education and decision-making must be a constant factor in programs/interventions for adolescents. Policies should encourage and support parents to openly discuss SRH topics with their adolescent children. Also, consideration should be given to subsidizing the costs associated with menstrual hygiene products, and comprehensive abortion care to make them more accessible. The dearth of technical skills in providing abortion services and long-acting contraceptives is a significant concern. For that reason, policies should prioritize training healthcare professionals to offer a wide range of SRH services tailored to adolescents' needs. This training should encompass not only medical expertise but also sensitivity to cultural nuances and ethical considerations.

### Strengths and limitations

The inclusion of a diverse range of stakeholders, including adolescents, parents, community leaders, and healthcare providers, enriched the study's insights. This diverse participant sample allowed for a holistic view of the complex dynamics surrounding adolescent SRH. Also, we followed an appropriate thematic framework in analyzing the data, thereby, facilitating the validity of the study. Furthermore, the findings of this study are consistent with many existing studies conducted in different jurisdictions. This suggest that the findings are transferable to similar sociocultural contexts. Due to the sensitive nature of SRH topics, participants might have provided responses that were socially desirable or aligned with perceived norms, leading to potential bias in their accounts. However, we tried to minimize this bias by including a diverse range of stakeholders.

## Conclusion

In conclusion, the findings from this study offer valuable insights into the complex landscape of adolescent sexual and reproductive health in the Bono East region. The implications for policy and practice are manifold, ranging from comprehensive education to addressing menstrual hygiene, involving parents, training healthcare providers, and promoting respectful care.

## Data Availability

The datasets generated and/or analysed during the current study are available upon reasonable request from the authors.

## Abbreviations

AHSPS: Adolescent Health Service Policy and Strategy
ARHP: Adolescent Reproductive Health Policy
LMICs: Low-and-middle-income Countries
SRH: Sexual and Reproductive Health
SSA: Sub-Saharan Africa
WHO: World Health Organization

## References

[1] Sawyer SM, Azzopardi PS, Wickremarathne D, Patton GC (2018). The age of adolescence. Lancet Child Adolesc Health.

[2] Bozzini AB, Maruyama JM, Santos IS, Murray J, Tovo-Rodrigues L, Munhoz TN, Matijasevich A (2023). Prevalence of adolescent risk behaviors at 11 and 15 years of age: data from the 2004 Pelotas birth cohort. Braz J Psychiatry.

[3] Sarkar A, Chandra-Mouli V, Jain K, Behera J, Mishra SK, Mehra S (2015). Community based reproductive health interventions for young married couples in resource-constrained settings: a systematic review. BMC Public Health.

[4] Gillespie B, Allen H, Pritchard M, Soma-Pillay P, Balen J, Anumba D (2022). Agency under constraint: adolescent accounts of pregnancy and motherhood in informal settlements in South Africa. Glob Public Health.

[5] Kyegombe N, Zuma T, Hlongwane S, Nhlenyama M, Chimbindi N, Birdthistle I, Floyd S, Seeley J, Shahmanesh M (2022). A qualitative exploration of the salience of MTV-Shuga, an edutainment programme, and adolescents’ engagement with sexual and reproductive health information in rural KwaZulu-Natal, South Africa. Sex Reprod Health Matters.

[6] Ahinkorah BO, Aboagye RG, Okyere J, Seidu AA, Budu E, Yaya S (2023). Correlates of repeat pregnancies among adolescent girls and young women in sub-Saharan Africa. BMC Pregnancy Childbirth.

[7] Sully EA, Biddlecom A, Daroch J, Riley T, Ashford L, Lince-Deroche N (2020). Adding It Up: Investing in Sexual and Reproductive Health 2019.

[8] World Health Organization (WHO). Adolescent Pregnancy: Key facts. 2023. Retrieved: https://www.who.int/news-room/fact-sheets/detail/adolescent-pregnancy. Accessed: 7 Aug 2023.

[9] Ghana Statistical Service (GSS), Ghana Health Service (GHS), and ICF International. Ghana Demographic and Health Survey 2014. Rockville, Maryland, USA: GSS, GHS, and. International ICF. 2015.

[10] Ghana Statistical Service (GSS), Ghana Health Service (GHS), ICF. Ghana maternal health survey 2017. Key Indicators Report Accra. 2018.

[11] Tenkorang EY, Amo-Adjei J, Kumi-Kyereme A, Kundhi G (2021). Determinants of sexual violence at sexual debut against in-school adolescents in Ghana. J Fam Violence.

[12] Boateng FD (2015). Victims of sexual assaults: The experiences of Ghanaian women. Int Rev Victimology.

[13] Yeboah I, Okyere J, Dey NE, Mensah RO, Agbadi P, Essiaw MN (2022). Multiple sexual partnership among adolescent boys and young men in Ghana: analysis of the 2003–2014 Ghana Demographic and Health Survey. Trop Med Health.

[14] Akumiah PO, Suglo JN, Sebire SY (2020). Early life exposures and risky sexual behaviors among adolescents: a cross-sectional study in Ghana. Niger Med J.

[15] Adam AR, Ganle JK, Asare BY, Baafi D, Letsa TS (2021). Risky sexual behaviours, contraceptive use and associated factors among unmarried female adolescents in an urban municipality in Ghana. Afr J Reprod Health.

[16] Kusi-Mensah K, Tamambang R, Bella-Awusah T, Ogunmola S, Afolayan A, Toska E, Hertzog L, Rudgard W, Evans R, Omigbodun O (2022). Accelerating progress towards the sustainable development goals for adolescents in Ghana: a cross-sectional study. Psychol Health Med.

[17] Rizvi F, Williams J, Maheen H, Hoban E (2020). Using social ecological theory to identify factors associated with risky sexual behavior in cambodian adolescent girls and young women aged 10 to 24 years: a systematic review. Asia Pac J Public Health.

[18] Republic of Ghana (2000). Adolescent reproductive health policy, 2000.

[19] Ghana Health Service (GHS). Adolescent Health Service Policy and Strategy (2016–2020); Ghana Health Service: Accra, Ghana, 2020; Available online: https://www.google.com/url?sa=t&rct=j&q=&esrc=s&source=web&cd=&ved=2ahUKEwiG3_zZydDyAhUmQEEAHYnzCRYQFnoECAQQAQ&url=https%3A%2F%2Fwww.afro.who.int%2Fsites%2Fdefault%2Ffiles%2F2017-10%2FADOLESCENT%2520HEALTH%2520SERVICE%2520POLICY%2520%2520AND%2520STRATEGY.pdf&usg=AOvVaw3bBY-GRD6_kQnXb-4Bno9A.

[20] Hall KS, Morhe E, Manu A, Harris LH, Ela E, Loll D, Kolenic G, Dozier JL, Challa S, Zochowski MK, Boakye A (2018). Factors associated with sexual and reproductive health stigma among adolescent girls in Ghana. PLoS One.

[21] Clarke V, Braun V, Hayfield N. Thematic analysis. Qualitative psychology: a practical guide to research methods. Qualitative psychology: a practical guide to research methods; Smith, JA, Ed. 2020:222–48.

[22] Bans-Akutey A, Tiimub BM. Triangulation in research. Acad Lett. 2021;2.

[23] Younas A, Fàbregues S, Durante A, Escalante EL, Inayat S, Ali P (2023). Proposing the “MIRACLE” narrative framework for providing thick description in qualitative research. Int J Qual Methods.

[24] General Assembly of the World Medical Association (2014). World Medical Association Declaration of Helsinki: ethical principles for medical research involving human subjects. J Am Coll Dent.

[25] Mbarushimana V, Conco DN, Goldstein S (2022). “Such conversations are not had in the families”: a qualitative study of the determinants of young adolescents’ access to sexual and reproductive health and rights information in Rwanda. BMC Public Health.

[26] Ahinkorah BO, Hagan JE, Seidu AA, Budu E, Hormenu T, Mintah JK, Sambah F, Schack T (2019). Access to adolescent pregnancy prevention information and services in Ghana: a community-based case-control study. Front Public Health.

[27] Okeke CC, Mbachu CO, Agu IC, Ezenwaka U, Arize I, Agu C, Obayi C, Onwujekwe O (2022). Stakeholders’ perceptions of adolescents’ sexual and reproductive health needs in Southeast Nigeria: a qualitative study. BMJ Open.

[28] Mohammed S, Larsen-Reindorf RE (2020). Menstrual knowledge, sociocultural restrictions, and barriers to menstrual hygiene management in Ghana: Evidence from a multi-method survey among adolescent schoolgirls and schoolboys. PLoS One.

[29] Asumah MN, Abubakari A, Aninanya GA (2022). Determinants of menstrual hygiene management practices among schoolgirls: a cross-sectional study in the Savannah Region of Ghana. Infect Dis Obstet Gynecol.

[30] Amo-Adjei J. Child Sexual Exploitation (CSE) in the Mining, Oil, Fishing, Agricultural, Construction and E-Waste (MOFACE) enclaves of Ghana. Accra: United Nations of Children’s Fund (UNICEF); 2022.

[31] Mason L, Nyothach E, Alexander K, Odhiambo FO, Eleveld A, Vulule J, Rheingans R, Laserson KF, Mohammed A, Phillips-Howard PA (2013). ‘We keep it secret so no one should know’–a qualitative study to explore young schoolgirls attitudes and experiences with menstruation in rural Western Kenya. PLoS One.

[32] Kyilleh JM, Tabong PT, Konlaan BB (2018). Adolescents’ reproductive health knowledge, choices and factors affecting reproductive health choices: a qualitative study in the West Gonja District in Northern region, Ghana. BMC Int Health Hum Rights.

[33] Gbagbo FY (2020). Profile of abortion seekers and their views on financial cost of induced abortions: implications for preventing unsafe abortions among young people in Accra. Ghana Public Health.

[34] Morgan AK, Agyemang S, Dogbey E, Arimiyaw AW, Owusu AF (2022). “We were girls but suddenly became mothers”: Evaluating the effects of teenage motherhood on girl’s educational attainment in the Volta Region. Cogent Soc Sci.

[35] Stoebenau K, Heise L, Wamoyi J, Bobrova N (2016). Revisiting the understanding of “transactional sex” in sub-Saharan Africa: a review and synthesis of the literature. Soc Sci Med.

[36] Wamoyi J, Ranganathan M, Kyegombe N, Stoebenau K (2019). Improving the measurement of transactional sex in Sub-Saharan Africa: a critical review. J Acquir Immune Defic Syndr..

[37] Tabong PT, Maya ET, Adda-Balinia T, Kusi-Appouh D, Birungi H, Tabsoba P, Adongo PB (2018). Acceptability and stakeholders perspectives on feasibility of using trained psychologists and health workers to deliver school-based sexual and reproductive health services to adolescents in urban Accra Ghana. Reprod Health.

[38] Mampane JN (2018). Exploring the “Blesser and Blessee” phenomenon: young women, transactional sex, and HIV in rural South Africa. SAGE Open.

[39] Amo-Adjei J, Kumi-Kyereme A, Tuoyire DA (2014). Transactional sex among female university students in Ghana: Implications for HIV education. Health Educ.

[40] UNICEF (2019). Right parent, right child [Electronic source]: Manual for positive parenting.

[41] Amo-Adjei J (2021). Toward an understanding of optimal grade for starting sexuality education programme for in-school children and adolescents: insights from Ghana. Am J Sex Educ.

[42] Nalwadda G, Mirembe F, Tumwesigye NM, Byamugisha J, Faxelid E (2011). Constraints and prospects for contraceptive service provision to young people in Uganda: providers' perspectives. BMC Health Serv Res.

